# A spatially explicit whole-system model of the lignocellulosic bioethanol supply chain: an assessment of decentralised processing potential

**DOI:** 10.1186/1754-6834-1-13

**Published:** 2008-07-28

**Authors:** Alex J Dunnett, Claire S Adjiman, Nilay Shah

**Affiliations:** 1 Centre for Process Systems Engineering, Department of Chemical Engineering, Imperial College London, London, SW7 2AZ, UK

## Abstract

**Background:**

Lignocellulosic bioethanol technologies exhibit significant capacity for performance improvement across the supply chain through the development of high-yielding energy crops, integrated pretreatment, hydrolysis and fermentation technologies and the application of dedicated ethanol pipelines. The impact of such developments on cost-optimal plant location, scale and process composition within multiple plant infrastructures is poorly understood. A combined production and logistics model has been developed to investigate cost-optimal system configurations for a range of technological, system scale, biomass supply and ethanol demand distribution scenarios specific to European agricultural land and population densities.

**Results:**

Ethanol production costs for current technologies decrease significantly from $0.71 to $0.58 per litre with increasing economies of scale, up to a maximum single-plant capacity of 550 × 10^6 ^l year^-1^. The development of high-yielding energy crops and consolidated bio-processing realises significant cost reductions, with production costs ranging from $0.33 to $0.36 per litre. Increased feedstock yields result in systems of eight fully integrated plants operating within a 500 × 500 km^2 ^region, each producing between 1.24 and 2.38 × 10^9 ^l year^-1 ^of pure ethanol. A limited potential for distributed processing and centralised purification systems is identified, requiring developments in modular, ambient pretreatment and fermentation technologies and the pipeline transport of pure ethanol.

**Conclusion:**

The conceptual and mathematical modelling framework developed provides a valuable tool for the assessment and optimisation of the lignocellulosic bioethanol supply chain. In particular, it can provide insight into the optimal configuration of multiple plant systems. This information is invaluable in ensuring (near-)cost-optimal strategic development within the sector at the regional and national scale. The framework is flexible and can thus accommodate a range of processing tasks, logistical modes, by-product markets and impacting policy constraints. Significant scope for application to real-world case studies through dynamic extensions of the formulation has been identified.

## Background

**Figure 1:**
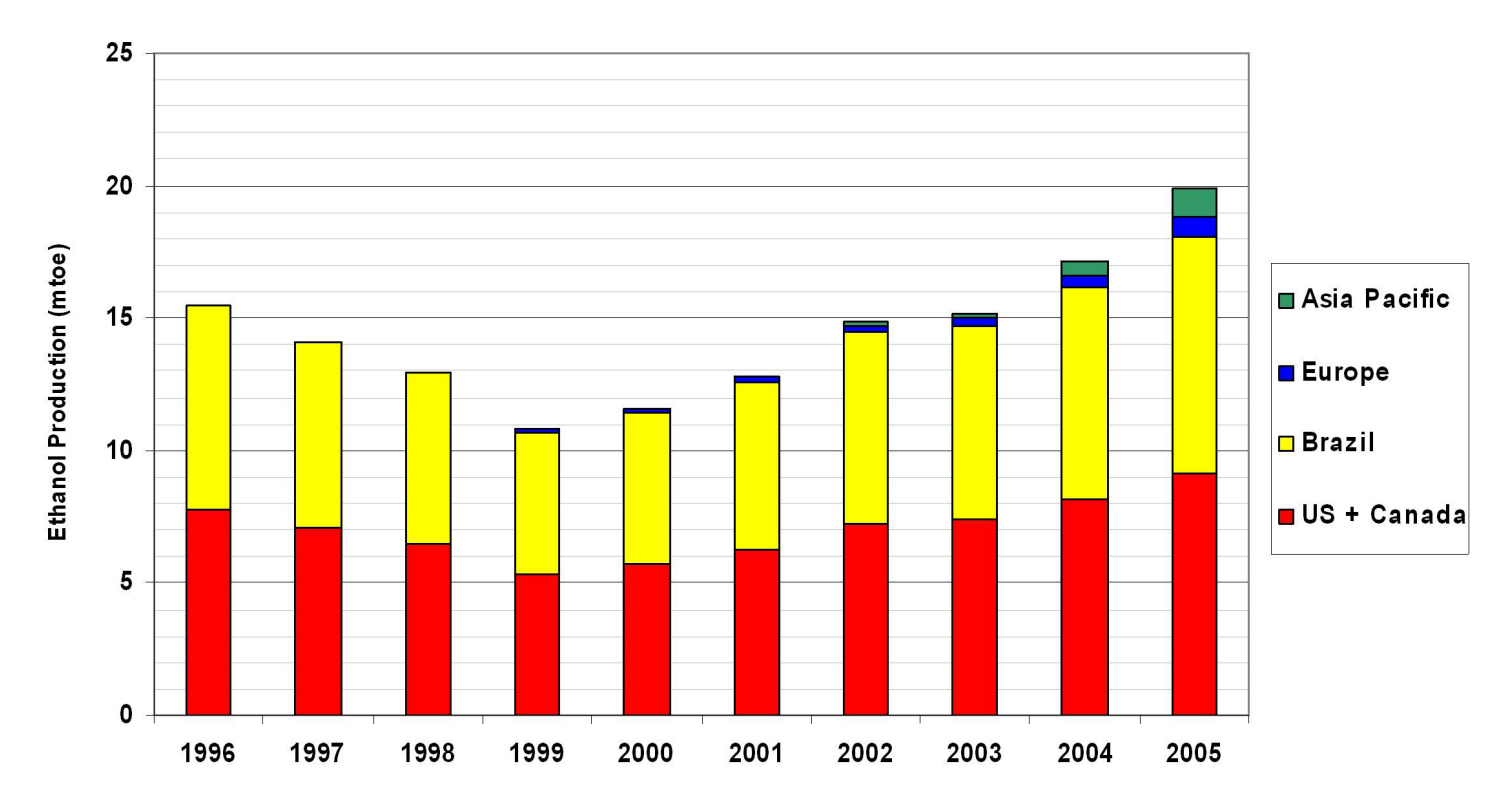
**Global bioethanol market trends 1996–2005**.

The penetration of biomass-derived ethanol (bioethanol) into the road transport fuels market has the potential to reduce greenhouse gas (GHG) emissions, improve fuel security, stimulate the agricultural sector and provide new markets for technology development and application. The 2006 global market for bioethanol was 20.2 million tonnes oil equivalent (mtoe), and was dominated by US and Brazilian production and consumption (45.4% and 43.9% of the total, respectively). Global growth (averaging 10.9% since 2001) has been fuelled predominantly through internal expansion of the juggernaut US and Brazilian programmes, but increasingly through expansion into the European and Asia-Pacific markets (see Figure 1 and [1]). This trend is expected to continue with the European Biofuels Directive targeting a 10% road transport fuel market share across the EU by 2020 (approximately 37 mtoe.yr^-1^, see [2]) in addition to an envisioned 30% replacement of current US petroleum consumption with biofuels by 2030 [3]. Bioethanol supply chains are anticipated to make a large contribution towards these targets through both domestic production and expanding international trade [4].

Current bioethanol production utilises so-called 'first-generation' technologies, processing sugar and hydrolysed starch crops using mature fermentation and separation processes. The effectiveness of these supply chains is the subject of much debate, and is constrained by the chain energetic efficiencies (ratio of primary energy inputs to derived ethanol high heating value (HHV), 0.79 MJ_PEI _MJ^-1^_EtOH_, see [5]), GHG abatement potential (reduction of net CO_2 _emissions per unit HHV substituted, 62.5 kg_CO2 _GJ^-1^_EtOH_, see [6]) and, most significantly, the availability of feedstocks that compete for land and agricultural market resources with food crops. Given land use concerns it is unlikely that US grain ethanol production, despite yield increases, will increase beyond three times the current production level [7].

Improved 'second-generation' pretreatment and fermentation technologies can alleviate this resource constraint through the utilisation of lignocellulosic (LC) biomass feedstocks. Diverse and abundant sources of LC biomass have been identified, including forestry and agricultural industry residues, dedicated energy crops and urban waste streams. The United Stated Department of Agriculture identified a potential 1.3 billion oven dry tonnes (odt) per year from US forestry and agriculture by the mid-21st century, requiring only modest changes in land use, agricultural and forestry practices [3].

LC-derived bioethanol is only recently starting to penetrate the global market. Its production currently involves the application of utility-intensive dilute acid or steam-explosion pretreatment and hydrolysis technologies to a range of 'residual' feedstocks, including wood chips, wheat straw, corn stover and bagasse. Scales of between 3000 and 30,000 m^3^_EtOH _year^-1 ^have been implemented at the pilot and demonstration scale, while early commercial plants of up to 200,000 m^3^_EtOH _year^-1 ^are expected to come online in 2008 (see [7]). These current technologies suffer from system interactions that result in a range of undesirable downstream impacts on enzymatic and microbial conversion efficiencies, leading to low-titre concentrations and subsequently high distillation energy requirements.

A document published by the US Genomics:GTL Program [8] recognises a high capacity for technological improvements in all aspects of the supply chain. These range from increases in the yield (15 to 25 odt ha^-1 ^year^-1 ^for high-yielding dedicated energy crops), stress tolerance and the lignin, cellulose, hemicellulose composition of energy crops, to enhanced pretreatment efficiency and greatly improved fermentation tolerance to titre concentration (40%_EtOH _is considered an optimistic target). Such advances could radically improve the process through enhanced lignin recovery for downstream electricity generation and reduced utility requirements, resulting in greater energetic efficiency and surplus utility availability. This would substantially increase net energy yields from agricultural land (Table 1). It is concluded that there exists substantial technological headroom for improvement of the LC-bioethanol system based on changes in agricultural biomass sources, pre-processing and fermentation microbial communities, with a trajectory towards consolidated bio-processing (CBP) in a single vessel [9].

**Table 1:** Performance metric comparison for starch- and LC-bioethanol life-cycles

	Metrics			
	
	GWP	Energy efficiency	Land productivity	Cost
Technology	kg_CO2 _GJ_F_^-1^	MJ_PEI _MJ_F_^-1^	GJ ha^-1 ^year^-1^	£_2006 _GJ^-1^

Starch	81	0.79	58	14.97
Lignocellulosic	11	0.1	94.5	8.41

Source	[5]	[5]	[29]	[30]

A question not considered in the literature is the resulting impact that such technological developments in LC-bioethanol feedstocks and processing technologies will have on the structure of the supply chain. Issues of facility location, economies of scale and logistical interconnection have been studied in the literature, albeit in the limited context of single plants encompassing all process steps (pretreatment, fermentation, separation, purification) taking place at one 'central' facility and utilising current technologies (that is, dilute acid hydrolysis [10-14]). Open questions remain, namely:

• What is the optimal configuration of multi-plant systems, areas of supply and demand and interconnecting logistics?

• Is there potential for process decentralisation through exploiting logistical cost gaps that arise from the large variation in material energy densities observed within current and future bioethanol supply chains?

The energetic density of biomass is low (3.0 to 4.6 GJ m^-3 ^for baled and chipped poplar, respectively), resulting in high logistics costs compared with pure/intermediate ethanol concentrations (the energy density of pure ethanol is 26.8 GJ m^-3^). These logistics costs act against the economies of scale available in conversion processes. This combination of factors may 'disrupt' the conventional perspectives on LC ethanol supply chains by making decentralised production infrastructures feasible, if not optimal. In such infrastructures, logistics costs would arise largely from the transport of high-energy density ethanol of intermediate purity.

The key goals of this work are:

• to develop a framework and methodology for assessing different spatial infrastructures of LC-bioethanol supply chains and their impact on system economics; and

• to investigate the evolution of the bioethanol supply chain with the development of dedicated energy crops and improved conversion technologies.

The next section details the methods applied in developing a model that characterises system economics, logistical flows and economies of scale in processing. The specific sources of data and assumptions underlying prescribed model parameters are also presented. We then present the results of an application of the model to a range of scenarios. These are followed by a detailed discussion regarding the limits of the modelling framework. The final section draws conclusions from the analysis presented.

## Methods

The LC-bioethanol supply chain system is assessed through the development of a spatially explicit model that combines production and logistics. This is based on the modelling approaches commonly applied in the optimisation of multi-site supply chain systems design [15,16] and operational planning [17]. The formulation builds on a model first developed and applied in the context of optimising future hydrogen infrastructures [18]. The model is formulated as a mixed-integer linear programming (MILP) model in GAMS [19] and solved to determine cost-optimal supply chain configurations. The modelling approach can be summarised as follows.

Given the following input data:

1) Spatial distribution of biomass supply

2) Spatial distribution of energy demand (ethanol, electricity, heat)

3) Material and energetic requirements of processing steps

4) Technology capital and operating costs

5) Distance, capacity and costs of biomass and ethanol logistics

6) Market structure

a. Hydrated or anhydrous ethanol market

b. Commodity market prices

Determine the optimal:

1) Regional purchase and supply strategy

2) Facility location

3) Facility scale and process-unit composition

4) Logistical interconnectivity and material flows

5) Production costs

The model formulation requires a large amount of information (input data) to be captured analytically within model parameters. The methods used to model process economics (including economies of scale), supply-demand distributions, logistics and processing performance are therefore presented. We also present mathematical formulations that are considered to have a significant impact on the model behaviour or that should be of specific interest to the reader. A concise summary of the full mathematical formulation is provided in Additional file 1. *Current *and *Future *scenarios are developed for feedstock and processing technology.

### Economics

Capital costs for both processing and logistics units are annualised through a periodic payment of total installed capital cost (*C*) as an annuity (*R*) as shown in Equation 1. A capital lifetime (*n*) is assigned specifically to each system component. A moderate discount rate (*i*) of 8% is assumed, representing the risk associated with the return on investment relative to an alternative allocation of capital. This figure is lower than that used by Kaylen et al [10] (15%) in order to represent the reduced risk in bioethanol investment anticipated under increased oil prices, and in line with increasing fiscal policy support for alternative energy technologies.

(1) $$R=C\left(\frac{i}{1-{\left(1+i\right)}^{-n}}\right)$$

Operating costs are assigned on an annual throughput basis (for example, in dollars per odt per year for raw materials). They account for required treatment-specific feedstocks (that is, enzymes, acids, denaturant), utilities (water, electricity, heat), labour, maintenance and overheads. Labour, maintenance and overheads are allocated between process components relative to fraction of total capital cost.

### Economies of scale

The economies of scale available in process unit capital and operating costs represent a key cost driver of the spatial system configuration, resulting in a preference (in the absence of other factors) for large, centralised facilities. Process plant economies of scale are typically captured through a continuous power law relating plant scale (*P*_1_) and capital cost (*C*_1_) through a scaling factor (α) relative to a base case with plant scale *P*_0 _and capital costs *C*_0_:

(2) $$\frac{P_{1}}{P_{0}}={\left(\frac{C_{1}}{C_{0}}\right)}^{\alpha }$$

Econometric studies are required to determine the scale factor (α). Hamelinck et al [9] and Wooley et al [14] identified the scaling factor for individual components for both current and future LC-bioethanol technologies. This facilitates disaggregation of the plant into specific processing steps in order to allow plant scale and process-unit composition at each location to be assessed. Capital cost scale factors are identified as 0.8 for alternative hydrolysis and fermentation technologies [9]. Economies of scale in operating costs were identified by Kaylen et al [10]. They identified the significant economies of scale available in administrative plant overheads (α = 0.25) compared with those operating costs linear with production (that is, α = 1 in the case of stoichiometric pretreatment treatment reagents).

### Spatial distributions

The hypothetical geographical area of study is discretised into a grid of homogeneous regions. We always use a 5 × 5 grid, that is, 25 regions. However, the size of each region is varied between 25, 50 and 100 km^2^. This allows the impact of local supply and demand distributions, system boundaries and the optimal configuration of multiple-plant infrastructures to be assessed at a range of length scales. For example, an optimal plant configuration identified at the 25 km^2 ^scale may not have access to sufficient easily accessible ('endogenous') resources in order to reach a truly 'optimal' plant scale. Furthermore, the optimum at the 25 km^2 ^scale may not remain optimal when the boundary is expanded to encompass a larger region containing additional plants. The optimal scale for assessment, balancing local spatial detail with global plant interactions, can therefore be identified as that scale which first approaches the minimum unit ethanol production cost (dollars per litre). This has important implications in spatially explicit infrastructure modelling wherein spatial resolution represents the dominant computational cost.

Hypothetical demand and supply scenarios are assigned through the specification of rural, semi-rural and urban land-cover types for each region. These are characterised by their agricultural land and population densities (Table 2). Values were derived from an assessment of the UK land-cover database [20] and regressed against population density derived from UK census data [21]. These values are therefore representative of UK and, more generally, European agricultural conditions. This does not affect the generality of the framework proposed here, as other regions can be considered by using different parameters. The discussion of the results, however, will necessarily be focussed on the UK and EU.

**Table 2:** Agricultural land cover and population densities for rural, semi-rural and urban region types

Region type	Agriculture (ha km^-2^)	Population (Capita km^-2^)
Rural	65	75
Semi-rural	25	300
Urban	5	1500

**Figure 2:**
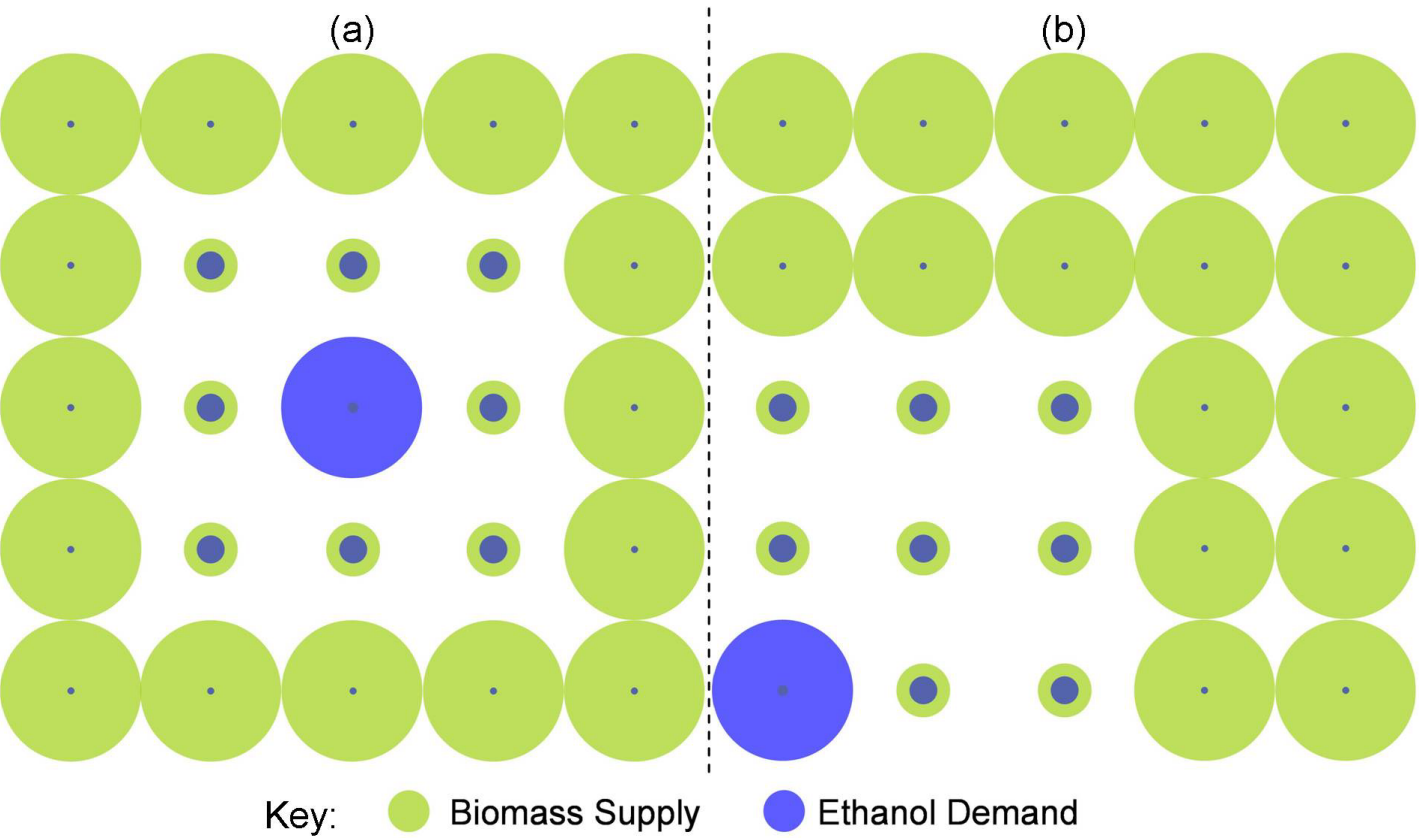
**Spatial distribution scenarios**. (a) Centralised. (b) Corner-Point. The size of the circles indicates the magnitude of the demand/supply.

Regional typologies are mapped onto the grid to generate the two 'generic' spatial distributions considered here. The *Centralised *distribution represents a central urban region with a peripheral semi-rural and rural boundary region. The *Corner-Point *distribution has the urban region located at the corner of the system, again with a peripheral semi-rural and rural boundary. This imposes a hard boundary on the urban demand epicentre, representative of a coastal or national border. These distributions are presented schematically in Figure 2.

### Feedstock supply

A 10% fractional availability of agricultural land for biomass sourcing is assumed, approximating the current EU set-aside quota. Feedstocks are characterised in terms of their lignin, hemicellulose and cellulose fraction and the higher heating value derived from the component fractions. The *Current *scenario process feedstock represents a generic crop residue such as wheat straw or corn stover. Harvested yield is assumed at 5 odt ha^-1 ^year^-1^. The *Future *scenario feedstock is assumed to represent a high-yielding hybrid poplar. Harvested yield is assumed at 25 odt ha^-1 ^yr^-1 ^for a three-year coppice cycle. A summary of feedstock properties is provided in Table 3. A farm gate commodity cost of $53.9 odt^-1 ^is assumed for both feedstocks (converted from UK cost data) in order to allow economies of scale and logistics cost drivers to be isolated.

**Table 3:** Feedstock properties

Property	Unit	Crop residue	Hybrid poplar
Yield	odt ha^-1 ^year^-1^	5.0	25.0
Cellulose	%_DM_*	36.4	44.7
Hemicellulose	%_DM_	22.6	18.6
Lignin	%_DM_	16.6	26.4
Inert mass	%_DM_	24.4	10.3
HHV	MJ.kg^-1^	15.2	18.5

* Percentage dry matter content determined on a wet basis

### Ethanol demand

Demand is assumed continuous at 2000 W per capita for electricity and heat and 980 W per capita for gasoline road-transport fuel demand [22]. Ethanol is assumed as a direct substitute for gasoline energy demand. The potential for heat provision from the ethanol refinery is limited to 10% of the regional heat demand, reflecting network installation and heat loss constraints in radial heat distribution.

The calculation of absolute regional demand requires subsequent allocation of per capita demand to total regional population, itself a function of population density and absolute spatial length scale. Population density was allocated in defining each of the three regional typologies (Table 2). Despite projected US and UK population increases of approximately 45% and 7% respectively by 2050, population growth is not considered when developing the *Future *scenario. The spatial distribution of demand, rather than its absolute magnitude, remains the dominant driver for optimal trade-offs in the system.

### Process technology

**Figure 3:**
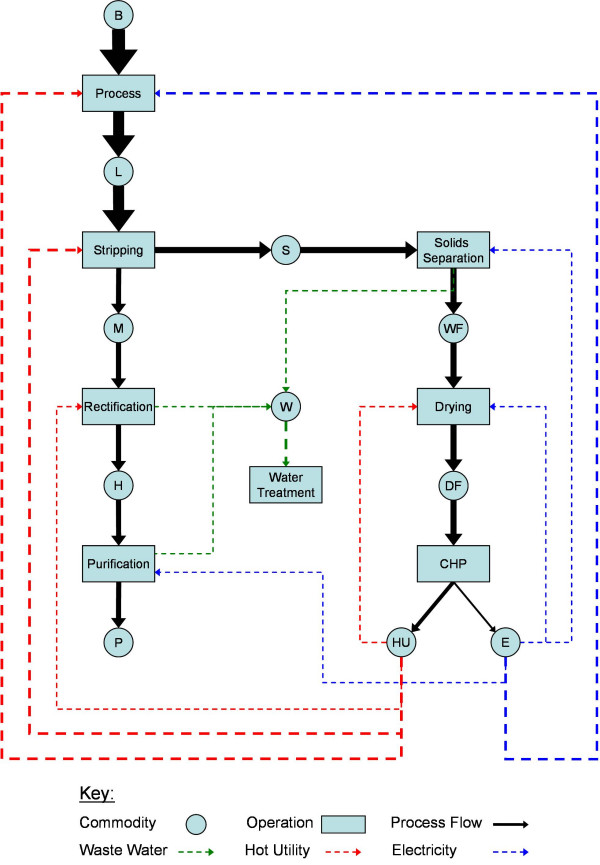
**A process flowsheet for the *Current *technology scenario**. The thickness of each arrow is representative of the relative energy content of that stream.

A generic process flowsheet for the LC-bioethanol production process is presented in Figure 3. This represents the network of feedstock, product and intermediate commodities, process technologies and their respective material and energetic interconnectivity. Incorporated process technologies and relative energetic flows are specific to the *Current *technology scenario.

The process flowsheet is composed of the pretreatment and fermentation process (*Process*), which generates a low-density ethanol titre (5.0wt%_EtOH_, L) from a biomass feedstock (B). The ethanol titre is concentrated through a purification train consisting of a stripping column (*Stripping*) to generate a medium-density intermediate (35.0wt%_EtOH_, M), a rectification column (*Rectification*) to generate the ethanol-water azeotrope (94.0wt%_EtOH_, H) and a membrane purification process (*Purification*) to generate the pure, anhydrous ethanol product (P). The *Future *scenario eliminates the need for the stripping step as fermentation titres are assumed to approach 35.0wt%_EtOH _through developments in microbial resistance to ethanol concentration. In addition, high titres via process intensification of fermentation (for example, fermentation with simultaneous ethanol stripping [23]) have already been demonstrated.

*Stripping *(or *Rectification *in the *Future *scenario) also generates a silage residue stream (S), which contains the unconverted cellulose and lignin fractions and the process water removed in the stripping column. This is passed to a solids separation unit (*Solids Separation*) which generates wet fuel (WF) and waste water (W) streams. The wet fuel is subsequently dried (*Drying*) to generate a dry fuel (DF) which is converted into hot-utility (HU) and electricity (E) in a combined heat and power unit (CHP).

The *Current *process technology is assumed to represent a single-stage saccharification and fermentation (SSF) process with pretreatment and fermentation conversion efficiencies of 75% and 95%, respectively. Overall conversion was assumed equal for both cellulose and hemicellulose fractions. The *Future *processing technology is envisaged to embody the principles of CBP with pre-processing and fermentation conversion efficiencies of 98% and 95%, respectively [9].

A transition to CBP technologies will significantly reduce the capital and operating costs of processing (that is, pre-processing, hydrolysis and fermentation) by an estimated 63% (see [9]). In optimal single-plant systems, increasing unit logistics costs balance against decreasing unit process and capital costs as the scale of the system increases (see [13] for a more detailed discussion). Thus, a reduction in capital and operating cost intensity, as embodied in the transition to CBP technologies, results in a downsizing of optimal single plants. This effect is countered in this work through the assumption of increased biomass yields per unit area in the *Future *scenario; this serves to reduce unit logistics costs.

### Energy integration

Feedstock composition affects the relative process energy flows (see Figure 3) through the respective allocation of feedstock HHV through pretreatment and fermentation efficiency, relative to each of the cellulose, hemicellulose and lignin fractions (assumed inert) to ethanol and residual fuel process streams. The resultant residual fraction is assumed to be combusted to provide a 25 bar steam input to a hypothetical Rankine cycle. This is designed to incorporate three pass-out turbines each generating power and steam utility at a specific pressure (11, 4 and 1 bar saturated steam). Turbine pressure ratios are scaled to match the internal process hot utility ratio requirements derived from [9]. Surplus electricity and heat represent valuable revenue streams.

As the front-end (pre-processing) of current processes require a large amount of heat, it is hard to decouple this from the back-end (utility generation), in particular because heat cannot be feasibly transported over large distances. Future pre-processing methods, identified by [9], apply steam explosion and compressed liquid hot water in order to hydrolyse the cellulose and hemicellulose fractions. These technologies continue to be hot utility intensive and therefore incompatible with *Process *decentralisation. The potential for the development of ambient processing is therefore explored. Proposed technologies include CO_2 _explosion [24], oxidative delignification (H_2_O_2_-catalysed enzymatic hydrolysis), and biological pretreatments (a concise review is provided by [25]). *Future *scenario *Process *hot utility requirements are therefore assumed negligible, substantially improving net energetic efficiency.

### Logistics

Logistics encompass all flows of mass and energy within the processing network. While this can be facilitated through pipeline or conveyor on site, it must be expanded to incorporate road, rail, pipeline and cable modes of transportation *between *sites (that is, located within different *regions*). Thus both *internal *and *external *process flows are characterised through a two-tier logistics network. Solid road transport using a 120 m^3 ^capacity trailer is assumed for feedstock biomass and both wet and dry residual fuels. Liquid road transport using 27 m^3 ^liquid tanker is assumed for dilute ethanol solutions (5%, 35% and 94% ethanol by weight) and pure ethanol. Rail logistics are not considered to be competitive owing to their high costs compared with road logistics over the relevant range of transport distances [26]. Pipeline transport is considered a feasible transport mode for all ethanol intermediate fractions, pure ethanol and wastewater. Heat is not considered mobile between regions, while electricity is assumed transported by existing electric cable at zero cost.

Logistics costs (*C*^*L*^) are modelled for each commodity in terms of duration (*C*^*T*^) and distance (*C*^*D*^) as

(3) $$C_{i,g,k}^{L}=C_{i}^{T}\left(\frac{2L_{g,k}\tau }{v_{i}}+LUT_{i}\right)+C_{i}^{D}2L_{g,k}\tau$$

The parameters in Equation 3 capture annualised capital, maintenance, labour and fuel costs and general overheads. Logistics costs specific to each commodity, mode of transport, source and destination are then a function of distance (*L*, assuming an empty return trip), the tortuosity of each mode (*τ*), transfer speed (*ν*) and total time spent loading and unloading (*LUT*). Here index *i *represents each specific commodity while *g *and *k *represent the source and destination region respectively. Logistics for biomass collection and ethanol distribution *within *each region are derived from an equivalent study completed for each region type.

### Intermediate purity ethanol logistics

In addition to the development of a framework for multi-plant infrastructure design, this work is focussed on assessing the potential for spatial decoupling of processes within the processing chain, resulting in distributed processing and centralised purification systems. The drivers for such behaviour can be characterised by two parameters: (1) the logistics ratio (LR)

(4) $$LR=\frac{{£}_{\text{Logistics}}m_{\text{EtOH}}^{-3}km^{-1}}{{£}_{\text{Logistics}}odt_{\text{Biomass}}^{-1}km^{-1}}$$

which represents the ratio between biomass and ethanol logistics costs (applicable at a range of purities); and (2) the economies of scale ratio (EoSR)

(5) $$EoSR=\frac{\alpha_{\text{Process}}}{\alpha_{\text{Purification}}}$$

which represents the ratio between the economies of scale factor (*α*) for front-end *Process *and downstream purification stages.

A decrease in *LR *can be achieved through the availability of pipeline technologies for both pure ethanol distribution (this is already standard practice in Brazil) and intermediate titres (that is, dilute 'crude' ethanol). The feasibility of pipeline distribution for slurries exhibiting solids concentrations of up to 30% on a wet basis was investigated by Kumar et al [27] for the case of corn stover transportation. Pipeline operating costs were assumed as $3.07× 10^-3 ^m^-3 ^km^-1 ^for pure ethanol [11] and $9.29 × 10^-2 ^odt^-1 ^km^-1 ^for a slurry representative of fermentation broths containing the residual lignin [27].

An increase in the EoSR can be envisioned to represent some degree of efficient downscaling and modularisation of the pretreatment and fermentation processes relative to downstream purification and utility generation. This would imply a shift in the capital and operating cost structure, in particular regarding labour and administrative overheads, such that costs are less dependent on scale. Such a scenario is also consistent with a supportive scheme of subsidies for small-scale producers, which would shift the balance of capital and operating costs within the processing system downstream.

### Model formulation

Commodity purchase, sale, processing and logistics are linked through a mass balance specific to each commodity within each region as illustrated in the following equation

(6) $$\begin{array}{l} \sum_{\begin{array}{l} \text{External}\\ \text{Regions} \end{array}}\left[\text{Logistics In}-\text{Logistics Out}\right]+\sum_{\text{Processes}}\left[\text{Generated}-\text{Consumed}\right]+\left[\text{Purchased}-\text{Sold}\right]=0\\ \text{for all}:\text{Commoditiies};\text{ Regions} \end{array}$$

The model is then solved in order to minimise total system logistics (both inter and intra-regional), process capital and operating costs:

(7) $$\begin{array}{l} \text{Minimise}\left[\sum_{\text{Regions}}\left(\text{Processing Costs}+\sum_{\text{Commodities}}\left[\text{Outbound Logistics Costs}+\text{Internal Logistics Costs}\right]\right)\right]\\ \text{for all}:\text{Demand Satisfaction Scenarios} \end{array}$$

A *Current *technology scenario is characterised as an SSF process utilising an agricultural residue feedstock and embedded within either a centralised or corner-point supply-demand distribution at the 50 km^2 ^grid scale. A *Future *technology scenario, employing an ambient CBP process utilising a hybrid poplar short rotation coppice (SRC) feedstock, is also considered. A summary of the technological parameters relevant to each scenario is provided in Table 4. Sensitivity to centralised and corner-point distributions, regional scale (25, 50 and 100 km^2 ^regions), and more detailed technological scenarios regarding logistics (Equation 4) and economies of scale (Equation 5) ratios are also explored.

**Table 4:** *Current *and *Future *scenario technology parameters

Parameter	Unit	Current	Future
Crop type	-	Straw residue	SRC hybrid poplar
Crop yield	odt ha^-1 ^year^-1^	5	25

Pretreatment method	-	Dilute acid	CO2 explosion
Process integration	-	SSF	CBP
Pretreatment conversion*	%_cellulose_	75.0	98.0
Fermentation conversion	%_sugars_	95.0	95.0
Ethanol yield**	l_EtOH _odt^-1^	281	382

Process titre	wt%_EtOH_	5.0	35.0
Process hot utility	MJ MJ_EtOH_^-1^	0.17	0.00
Distillation hot utility***	MJ MJ_EtOH_^-1^	0.26	0.20

*Assumes equal conversion of both cellulose and hemicellulose fractions

**Pure ethanol product

***Distillation includes both *Stripping *and *Rectification *stages for the *Current *technology

## Results

**Figure 4:**
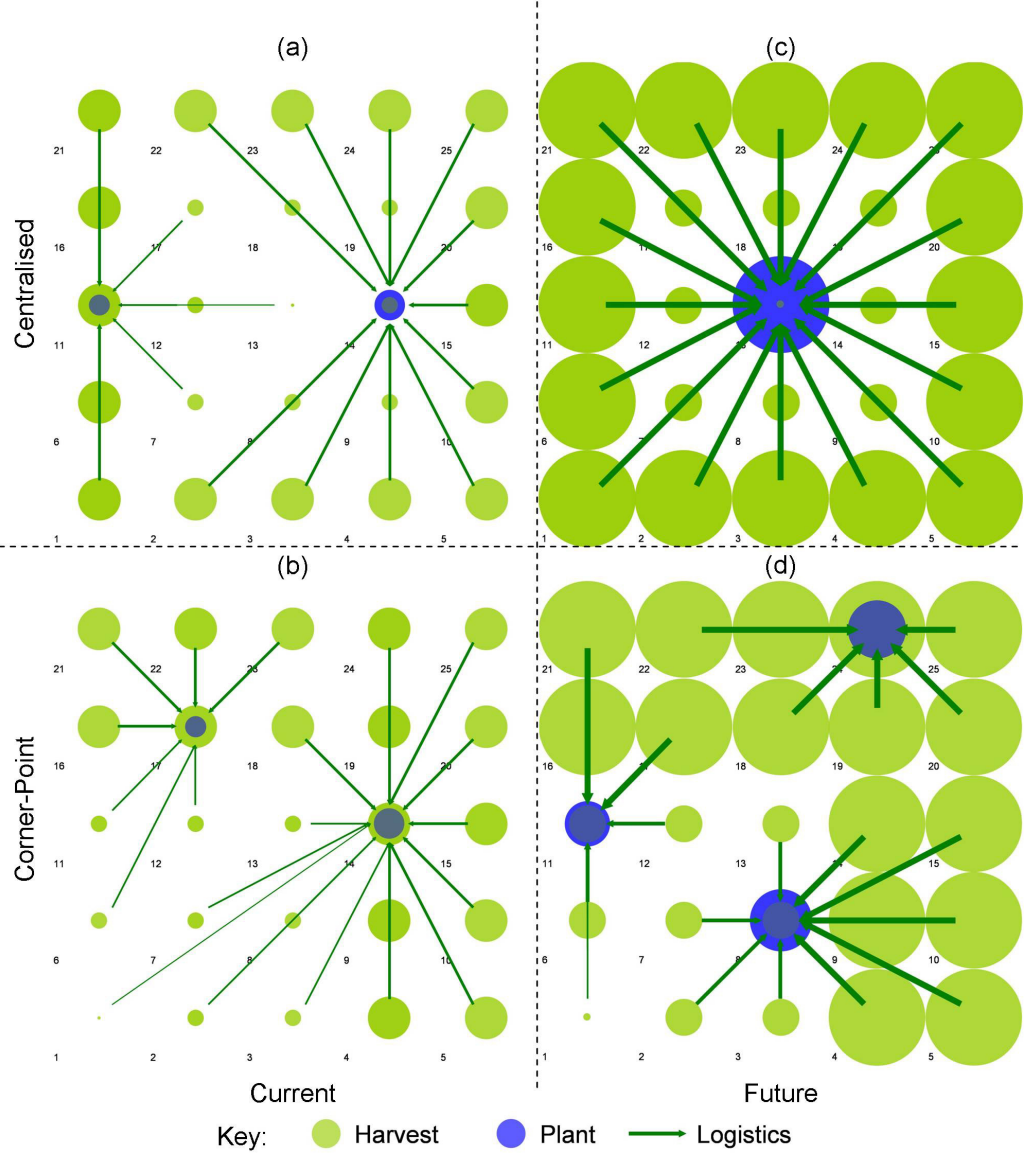
**System configurations at the 50 × 50 km^2 ^region scale**. Refer to Figure 2 for the underlying supply and demand distributions. The relative scale of harvesting is represented by the disk *radius*. The relative scale of process operations is represented by disk *area*. This scheme was selected to enhance visual clarity.

Cost-optimal configurations illustrating optimal plant location and biomass sourcing at the 50 km^2 ^scale are presented and compared in Figure 4. Ethanol distribution logistics do not represent a significant driver of the spatial system owing to a comparatively low cost and therefore are not presented. A number of system performance metrics are provided for each spatial distribution, scale and technology scenario in Table 5.

**Table 5:** System performance metrics for spatial scale, spatial distribution and technological scenarios

Scenario			Current			Future		
			
			Grid scale (km^2^)			Grid scale (km^2^)		
	
	Metric	Unit	25	50	100	25	50	100
Centralised	Ethanol cost	$_2007_.l_EtOH_^-1^	0.714	0.605	0.579	0.350	0.328	0.328
	${{\bar{L}}_{B}}^{*}$	km	53	70	114	53	107	97
	L:C ratio**	-	0.17	0.29	0.45	0.45	0.86	0.79
	No. plants	-	1	2	4	1	1	5
	Max. plant***	10^6 ^l year^-1^	109	297	546	742	2970	2620

Corner-Point	Ethanol cost	$_2007 _l_EtOH_^-1^	0.712	0.601	0.578	0.355	0.337	0.346
	${{\bar{L}}_{B}}^{*}$	km	48	71	102	48	66	82
	L:C ratio	-	0.17	0.28	0.46	0.33	0.49	0.75
	No. plants	-	1	2	4	1	3	8
	Max. plant	10^6 ^l year^-1^	109	297	533	742	1230	2380

*The average unit-biomass transport distance

**The ratio between logistics costs and process capital and operating costs

***Maximum single-plant scale observed within system

The *Current *technological system splits into 141 × 10^6 ^l year^-1 ^and 297 × 10^6 ^l year^-1 ^capacity plants for both spatial scenarios (Figures 4a and 4b). The average biomass transport distance (${\bar{L}}_{B}$) is 83.7 km with a maximum range of 140 km. A higher spatial resolution would allow a more accurate maximum range to be determined.

The higher biomass yield in the *Future Centralised *scenario (Figure 4c) supports a larger plant(s) for the same sourcing footprint. A single plant of 2.97 × 10^9 ^l year^-1 ^is observed, sourcing biomass over an average distance of 106.9 km. Whilst this appears large by current standards, it represents a plant of approx. 2.2 GW ethanol output capacity, comparable with small petrochemical refining operations. This is the only scenario tested which complies with the optimal L:C Ratio of approximately 0.8 as derived by Wright and Brown [13] for single-plant systems (Table 5). Therefore this metric is not considered a robust indicator of optimality for systems of multiple plants located within heterogeneous biomass supply distributions.

The future technology appears more sensitive to the spatial distribution scenario. In the corner-point case (Figure 4d) a three-plant system is observed comprising a range of smaller plant capacities of 0.66, 1.085 and 1.23 × 10^9 ^l year^-1^. The average biomass transport distance is reduced to 66.1 km. It would appear that a shift to high-yielding energy crops and CBP drives an increased sensitivity to the spatial distribution of biomass and, to a lesser extent, ethanol demand. This suggests that ethanol distribution becomes a factor. Note that while spatial structure and L:C ratio differ greatly, the cost of ethanol production remains very close.

**Figure 5:**
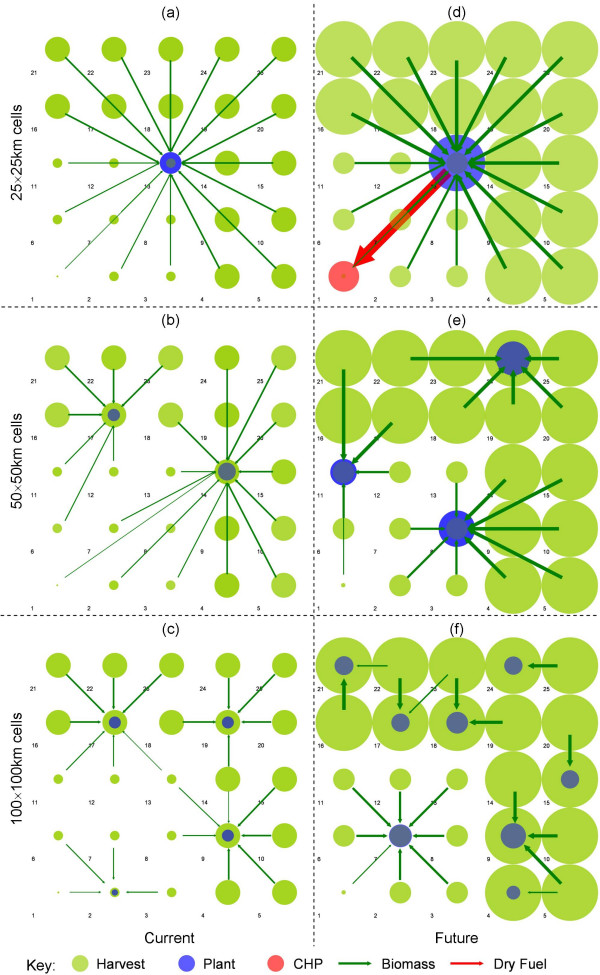
**System configurations for the *Corner-Point *distribution**. Refer to Figure 2b for the underlying supply and demand distribution.

The sensitivity of the corner-point distribution to the spatial scale of the system provides further insight into the influence of technological development on the optimal plant configuration and logistical flows. These are presented in Figure 5.

At the 25 km^2 ^scale, the single, centrally located plant configuration dominates (Figures 5a and 5d). This location minimises the average unit biomass transportation distance to the single plant. The increased lignin yield in a transition to a hybrid poplar feedstock and the significant decrease in process and separation hot utility requirements results in an excess potential hot utility generation (limited to 10% of potential regional demand) for the *Suburban *plant location region. As a result, a CHP plant is established supplying 48 MW_e _and 59 MW_th _within the high population density urban region (grid 1).

For both technological scenarios at the 100 km^2 ^scale (Figures 5c and 5d) a dedicated plant is located within the sparse suburban region while a number of plants compete for biomass resource throughout the rural periphery. Observed plant scales range between 150 and 540 × 10^6 ^l year^-1 ^for the *Current *and 1.24 and 2.38 × 10^9 ^l year^-1 ^for the *Future *scenario. The average biomass transport distance is 101.9 km for the *Current *scenario and 81.6 km for the *Future *scenario.

System performance metrics are presented in Table 5 for each of the tested scenarios. For both technological scenarios, there is negligible sensitivity in ethanol production cost to spatial distribution. The *Current *technology exhibits a clear reduction in production cost with increasing spatial scale owing to a significant increase in available resource and thus achievable economies of scale in production. This effect is less pronounced in the *Future *scenario owing to a proportional increase in biomass costs and an apparent trend to the optimal scale for system operation, as indicated by the minimum in production costs for the *Corner-Point *scenario at a scale of 50 km^2^.

### Intermediate purity ethanol logistics

The potential for systems involving the logistics of crude, intermediate concentration ethanol has been investigated through an assessment of sensitivity to increases in the Logistics Ratio (LR, Equation 4) through the incorporation of pipelines for the movement of both pure and intermediate ethanol concentrations. These have been tested for the *Future *scenario at the 50 km^2 ^grid scale, as this was identified as close to the production cost optimum (Table 5).

**Figure 6:**
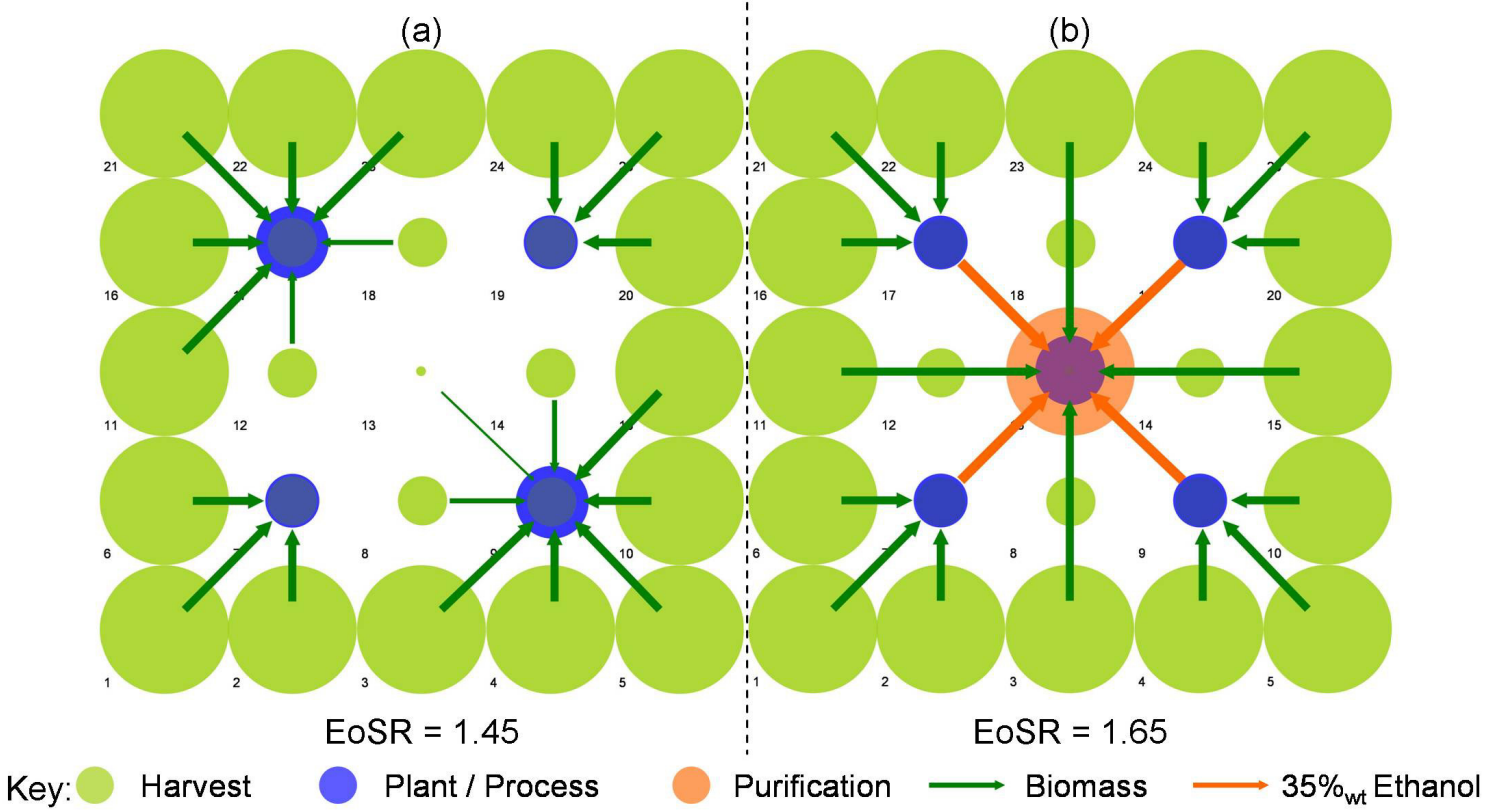
**System configurations for the economies of scale ratio scenarios at the 50 × 50 km^2 ^region scale**. Refer to Figure 2a for the underlying supply and demand distribution.

The base-case EoSR for *Future *technologies is 1.28. Scenarios driving EoSR to 1.45 and 1.65 have been investigated. Figure 6 presents the EoSR scenarios for the *Centralised *distribution. Figure 6a demonstrates that a small reduction in the economies of scale in *Process *operations drives the optimal system configuration from the centralised system observed in Figure 4c to one encompassing multiple, smaller-scale facilities, ranging between 525 × 10^6 ^l year^-1 ^and 954 × 10^6 ^l year^-1^, located within the suburban boundary.

The scenario in which EoSR = 1.65, observed in Figure 6b, assumes upper and lower bound scaling factors of 0.92 and 0.55 for *Process *and combined *Purification *and *Utility *respectively. This results in the centralisation of purification and utility generation whilst biomass processing to intermediate ethanol concentrations is decentralised within the suburban periphery. The intermediate ethanol titre is transported between sites via liquid tanker.

The introduction of pipeline technologies promotes decoupling and decentralisation of *Process *operations (Figure 6b) for both EoSR = 1.45 and 1.65 scenarios. This structural adaptation in the case of the EoSR = 1.45 scenario is driven through a significant reduction in pure ethanol logistics costs through pipeline, rather than liquid tanker, transport. Previously, economies of scale in centralised *Purification *and *Generation *were outweighed by increased ethanol distribution costs to the *Suburban *and *Rural *periphery. This is the first observed case wherein ethanol distribution logistics are clearly driving the optimal system configuration. A modest reduction in ethanol production costs of 3.3% is observed through the introduction of pipeline transport of pure ethanol for the *Future Centralised *scenario. Details of system metrics for each of the EoSR and ethanol transport mode scenarios are outlined in Table 6.

**Table 6:** System performance metrics for economies of scale ratio and logistical mode scenarios

			Liquid transport mode	
EoSR	Metric	Units	Tanker	Pipeline

1.28	Ethanol cost	$_2007 _l_EtOH_^-1^	0.328	0.317
	${{\bar{L}}_{B}}^{*}$	km	107	107
	L:C ratio**	-	0.86	0.76
	No. plants	-	1	1
	Configuration	-	Integrated	Integrated

1.45	Ethanol cost	$_2007 _l_EtOH_^-1^	***	-
	${\bar{L}}_{B}$	km	58	62
	L:C ratio	-	0.34	0.58
	No. plants	-	4	5
	Configuration	-	Integrated	Decentralised

1.65	Ethanol cost	$_2007 _l_EtOH_^-1^	-	-
	${\bar{L}}_{B}$	km	62	62
	L:C ratio	-	0.69	0.59
	No. plants	-	5	5
	Configuration	-	Decentralised	Decentralised

*The average unit-biomass transport distance

**The ratio between logistics costs and process capital and operating costs

***Production costs for EoSR scenarios are not considered valid owing to distortions of relative and absolute component costs

The sensitivity to the negligible *Process *heat assumption for the *Future *technology, resulting from the development of ambient processing methods, was assessed for the EoSR = 1.65 scenario. A hot utility requirement equivalent to 30% (per odt of biomass processed) of that required for the *Current *scenario was assumed. The resulting system abandons ethanol intermediates and switches to a system of decentralised, fully integrated plants similar to that observed in Figure 6a.

## Discussion

The model results presented provide insight into the optimal system configurations for a range of potential scenarios in response to economies of scale and logistical factors. However, the model neglects the impacts of the system dynamics on system performance. The conceptual model should therefore be expanded with a discussion of dynamic factors applicable to the operational, planning and strategic timeframes. Opportunities for future work are presented.

Storage-related issues, such as storage location, and its role in dictating supply chain structure, have been neglected within the model. This is because storage does not have an influence on the spatial structure of the system in a steady-state model with known demands at each location. Assuming that constant operational profiles are desirable, because they minimise underutilised process capital, the drivers of biomass storage location reduce to two contributing factors: economies of scale in storage, and the degradation rate of biomass. It is clearly undesirable to incur monetary and energetic cost transporting biomass that will degrade prior to processing. An assessment of biomass storage location would therefore require an extension of the modelling framework presented to consider dynamics at the monthly or seasonal temporal resolution. Furthermore, it would require consideration of an expanded range of pretreatment and densification operations that impact on biomass energy density and propensity to microbial degradation. This will be considered in future work.

In order to provide some insight into the scale of biomass storage required it is noted that the capacity required to house the total annual harvest within each rural region for the *Future *scenario at 50 km^2 ^scale would be of the order of 2.7 × 10^6 ^m^3^. Three-day buffer storage at a central plant, at the extreme scales of operation observed in, for example, the 2.9 × 10^9 ^l year^-1 ^plant, requires approximately 46 × 10^3 ^m^3 ^of shed capacity. Furthermore, the total number of truck deliveries per day becomes a factor with regard to plants located in an urban region. At the suggested optimal scale, 50 deliveries per hour would be required. Constraints on feasible logistical operations could potentially become apparent well below this threshold.

The assumption of negligible hot utility requirement in *Future Process *operation, achieved through the application of CO_2 _explosion and oxidative delignification, is considered highly optimistic. It is nevertheless posited within the cost assessment presented, in an attempt to incorporate potential new processes, as identified through the US Department of Energy Genomics:GTL Program [8]. The target of such processes is a shift away from relatively harsh thermochemical treatments through the identification of biological (microbial and enzymatic) pathways capable of reducing pretreatment severity. Solutions are envisaged in: (1) the genetic engineering of LC cell-wall structures to be more receptive to bio- and thermochemical treatments; (2) upstream processing during storage (ensilage); and (3) the development of 'ligninases', providing an enzymatic basis of lignin depolymerisation. The optimal configuration of biological pretreatment processes throughout the supply chain presents a fascinating challenge that requires open-minded consideration prior to innovation in order to prevent 'tunnel-vision' approaches with limited potential benefits.

It can be argued that, as a result of a transition towards ambient pre-processing, the reaction rates achieved through intensive thermochemical treatments cannot be maintained. However, the principles of distributed processing are consistent with reduced reaction rates owing to the relaxation of time constraints in batch processing, wherein the shipping of ethanol of intermediate concentration can be scheduled to match batch process completion. As such, ambient processing could provide both production and storage capacity within a multi-site production, storage and collection schedule, the total vessel capacity being approximately the same in each case. A dynamic, combined production and logistical scheduling formulation of the model developed here would provide insight into the economic potential of such a system (see, for example, [28]).

A dynamic formulation, at a seasonal or monthly resolution, would facilitate the investigation of the role of heat market interactions on the residual lignin treatment chain. This work assumes that all lignin is combusted within a CHP facility. Whilst representing the net energetic optimum for the system, with processing chain efficiencies for the *Current *and *Future *technologies of 44.4% and 66.8%, respectively, this has a significant impact on the process economics of plants at smaller scales. This is observed in the large range of ethanol production costs for the *Current *technology in Table 5. Indeed, the range of options for lignin treatment in pre-processing, downstream separation and eventual disposal/conversion requires further investigation owing to the significant impact on net chain energy efficiency. The lignin treatment chain also exhibits substantial feedback through system interactions regarding pretreatment severity and distillation energy requirements. Further assessment incorporating the option of dedicated combustion plants is considered. These could be more readily decentralised, compared with fully integrated CHP systems, reducing the sensitivity of decentralised processing configurations (Figure 6b) to *Process *hot utility requirements.

This work attempts to capture strategic dynamics (5 to 25 years) through the assessment of a steady-state model within 'snapshot' technological scenarios. In reality, the transition from the current to the future state provides many challenging decisions regarding plant construction, plant shut-down and retrofit planning, and logistical infrastructure investment. Ensuring that (near-)optimal systems are achieved in the future hinges on whether optimal current configurations are robust under future conditions and on the migration pathway between the technologies that is anticipated.

This work suggests that the general form of optimal configurations is robust for both *Current *and *Future *technological scenarios. While a five-fold increase in feedstock yield alone would drive a significant increase in optimal plant scale, this effect is tempered by a concurrent reduction in process capital and operating costs relative to logistics. Wright and Brown [13] provide a simple analytic model to test this result for isolated single-plant systems. In the case of multiple-plant infrastructures, locating and scaling the 'first' plant without consideration of the globally optimal system configuration under both current and future conditions could result in a legacy of system-wide sub-optimal performance extending throughout the lifetime of the plant. The model applied in this work therefore presents an invaluable tool in the strategic design of LC-bioethanol supply chain systems. A dynamic capacity start-up and shut-down extension of model framework will be developed and applied in future work.

## Conclusion

A spatially explicit whole-systems assessment of current and possible future LC-bioethanol infrastructures has been completed. Hypothetical future development scenarios (within the bounds of scientific and engineering postulates) for agricultural, processing and logistical (pipeline) technologies were applied in order to gain some insight into the potential evolution of the future infrastructure. Current technologies were characterised by agricultural residue feedstocks (for example, corn stover), dilute-acid hydrolysis and single-stage SSF. Future technologies were characterised by high-yielding energy crops (for example, hybrid poplar), a transition towards ambient pre-processing technologies and CBP.

Optimal ethanol production costs for current technologies are highly sensitive to the spatial scale of the assessment. They decrease significantly from $0.71 to $0.58 per litre concurrent with increasing economies of scale in processing up to a limiting plant scale of 550 × 10^6 ^l year^-1^. Future feedstocks and technologies realise significant cost reductions with production costs ranging from $0.33 to $0.36 per litre. Cost-optimal future systems were observed to be increasingly sensitive to the spatial distribution of biomass supply.

The potential for decentralised production systems involving the logistics of crude, intermediate concentration ethanol has been considered. The large hot utility requirements of current pre-processing technologies and ethanol distillation stages prevent decoupling of front-end processing from the highly capital intensive, tail-end lignin treatment and utility generation. Future increases in feedstock yields repress the driver for front-end process decentralisation as they support larger, fully integrated plants over the same biomass sourcing footprints.

An increase in the ratio of economies of scale factor between front-end processing (that is, pretreatment and fermentation) and downstream separation technologies was considered, embodying some degree of efficient downscaling and modularisation of the pretreatment and fermentation processes. At scale factor ratios of 1.45 and above, distributed front-end process systems were observed. However, these must be considered as highly sensitive to the assumption of ambient pretreatment technologies. The incorporation of pipelines as a feasible mode for transport of intermediate and pure ethanol titres had a limited impact on whole-system economics and spatial configuration.

The modelling approach and formulation presented provide a valuable analytical tool for the optimisation of the spatial LC-bioethanol supply chain. In particular, it can provide insight into the optimal configuration of multiple-plant systems. This information is invaluable in ensuring (near-) cost-optimal strategic development within the sector at the regional and national scale. The framework is flexible and can thus accommodate a range of processing tasks, logistical modes, by-product markets and impacting policy constraints (obligations, subsidies). There exists great scope for application to real-world case studies through dynamic extensions of the formulation.

## List of abbreviations

CBP: Consolidated bio-processing; CHP: Combined heat and power; DM: Dry matter; EoSR: Economies of scale ratio; F: Feedstock; GHG: Greenhouse gas; HHV: Higher heating value; LC: Lignocellulosic biomass; LR: Logistics ratio; MILP: Mixed Integer Linear Programming; ODT (odt): Oven-dry tonne(s); PEI: Primary energy input(s); SRC: Short rotation coppice; SSF: Simultaneous saccharification and fermentation.

## Authors' contributions

AJD completed the majority of the work with regard to the system study, mathematical model development, results analysis and drafting of the manuscript. Both CSA and NS had substantial input into the structure and content of the manuscript. NS conceived of the study, steered its direction and provided support regarding computational issues. All authors read and approved the final manuscript.

## Supplementary Material

Additional file 1 Details of the mathematical model formulation. Click here for file
