# Supplementary material for: A spatially explicit whole-system model of the lignocellulosic bioethanol supply chain: an assessment of decentralised processing potential
Source: Biotechnol Biofuels. 2008 Jul 28;1:13. doi: 10.1186/1754-6834-1-13 (PMC2546396; doi:10.1186/1754-6834-1-13)
Supplement: Additional file 1 — Details of the mathematical model formulation. [file 1754-6834-1-13-S1.pdf]

Model Formulation

The mathematical model formulation is presented. This represents a general model formulation with no specific application implied.

Nomenclature

Sets

$g \in G$	set of regions
$i \in I$	set of commodities
$j \in J$	set of technological scales
$k \in G$	alternative reference for set of regions
$m \in M$	set of logistics modes
$p \in P$	set of technologies
$I^D \subseteq I$	subset of demand satisfaction commodities
$FM_{m,i} \subseteq M \quad \forall i \in I$	subset of feasible logistics modes m for each commodity i
$L^A_{g,k} \subseteq G \quad \forall g \in G$	subset of adjacent regions k relative to each region g

Parameters

$A_{i,g}$	availability of commodity i in region g
C_i	market price of commodity i
$CB_{p,j}$	cost upper bound for technology p at scale j
$DM_{i,g}$	demand for commodity i in region g
DW_m	drivers wage for logistics mode m
FE_m	fuel economy for logistics mode m
FP_m	fuel price for logistics mode m
GE_m	general expenses for logistics mode m
$IT_{i,g}$	internal transfer cost for commodity i in region g

$L_{g,k}$	distance between region g and region k
LUT_m	total loading and unloading duration for logistics mode m
$M1$	maximum regional surplus
$M2$	maximum internal transfer cost
ME_m	maintenance expenses for logistics mode m
OP_m	operating period of logistics mode m
PP_m	payback period of logistics mode m
SP_m	average speed of logistics mode m
$TCap_{i,m}$	capacity of logistics mode m in carrying commodity i
TMA_m	availability of logistics mode m
TMC_m	capital cost of logistics mode m
$VB_{p,j}$	upper bound capacity for technology p at scale j
$\rho_{i,p}$	relative commodity i input to technology p
$\bar{\rho}_{i,p}$	relative commodity i output from technology p
τ_m	tortuosity of logistics mode m

Continuous Variables

$CIT_{i,g}$	internal transfer cost for commodity i in region g
CPC	total commodity purchase cost
$CV_{p,g}$	total cost of technology p in region g
$D_{i,g}$	total commodity i sold in region g
FC	fuel cost
GC	general costs
LC	labour cost
MC	maintenance cost
PCC	total process capital and operating cost
$Q_{i,m,g,k}$	flow of commodity i via mode m from region g to region k

$R_{i,g}$	total commodity i purchased in region g
REV	total commodity sales revenue
TCC	total logistics capital cost
$TCIT$	total internal transfer cost
TOC	total logistics operating cost
$V_{p,g}$	capacity of technology p in region g
$VI_{j,p,g}$	capacity of technology p at scale j in region g

Integer Variables

NTU_m	number of units of logistics mode m selected

Binary Variables

$Y_{i,g}$	import decision for commodity i in region g
$\delta_{j,p,g}$	selection of technology p at scale j in region g

Equations

The model equations are presented here in a modular form for the sake of clarity. A brief description of the equation set is presented for each module.

Logistics Module

$$\sum_{g}\sum_{k} Q_{i,m,g,k} = 0 \qquad \forall i \in I, m \notin FM_{m,i}$$

Eqn.A1

$$\sum_{i}\sum_{m} Q_{i,m,g,k} = 0 \qquad \forall g \in G, k \notin L^{A}_{g,k}$$

Eqn.A2

$$NTU_m = \sum_{i}\sum_{g}\sum_{k}\left(\frac{Q_{i,m,g,k}}{TMA_m.TCap_{i,m}}\right)\cdot\left(\frac{2.L_{g,k}.\tau_m}{SP_m} + LUT_m\right) \qquad \forall m \in M$$

Eqn.A3

$$FC = \sum_{i}\sum_{m}\sum_{g}\sum_{k} FP_m \left(\frac{2.L_{g,k}.\tau_m.Q_{i,m,g,k}}{FE_m.TCap_{i,m}} \right)$$

Eqn.A4

$$GC = \sum_{m} GE_m.NTU_m$$

Eqn.A5

$$LC = \sum_{i}\sum_{m}\sum_{g}\sum_{k} DW_m \left(\frac{Q_{i,m,g,k}}{TCap_{i,m}} \right) \cdot \left(\frac{2.L_{g,k}.\tau_m}{SP_m} + LUT_m \right)$$

Eqn.A6

$$MC = \sum_{i}\sum_{m}\sum_{g}\sum_{k} ME_m \left(\frac{2.L_{g,k}.\tau_m.Q_{i,m,g,k}}{TCap_{i,m}} \right)$$

Eqn.A7

$$TCC = \sum_{m} \frac{NTU_m.TMC_m}{OP_m.PP_m}$$

Eqn.A8

$$TOC = LC + FC + MC + GC$$

Eqn.A9

Eqn. A1 constrains inter-regional logistics to those modes feasible for each commodity. Eqn. A2 constrains inter-regional logistics to only those adjacent regions (1) preventing looping of material within a region and (2) reducing the number of active, and computationally expensive, logistics flow variables. Eqn. A3 assigns the number of logistical units required. The integer variable (NTU_m) can be relaxed in order to reduce computational complexity. This results in the linear formulation presented in the full text (Eqn. 3). Eqn. A4-A7 assigns logistical costs relative to distance and time specific parameters. Eqn. A8 allocates total unit capital cost relative to the time period of study. Eqn. A9 summates the total logistical operating costs.

Internal Logistics Module

$$-M1.Y_{i,g} \le \sum_{p} V_{p,g}.\left(\overline{\rho}_{i,p} - \rho_{i,p}\right) + R_{i,g} - D_{i,g} \le M1.\left(1 - Y_{i,g}\right) \quad \forall i \in \mathrm{I}, g \in G$$

Eqn.A10

$$CIT_{i,g} \geq \left(\sum_{p} V_{p,g} . \rho_{i,p} + D_{i,g} \right) . IT_{i,g} - Y_{i,g} . M2 \quad \forall i \in \mathrm{I}, g \in G$$

Eqn.A11

Net-importing region commodity constraint

$$CIT_{i,g} \geq \left(\sum_{p} V_{p,g} . \overline{\rho}_{i,p} + R_{i,g} \right) . IT_{i,g} - \left(1 - Y_{i,g}\right) . M2 \quad \forall i \in \mathrm{I}, g \in G$$

Eqn.A12

Total internal logistics cost summation

$$TCIT = \sum_{i} \sum_{g} CIT_{i,g}$$

Eqn.A13

Eqn. A10 allocates the decision to import variable ($Y_{i,g}$) with respect to regional commodity surplus or deficit. Eqn. A11-A12 determines the relative internal logistics costs, driven by cost minimisation objectives (See below, Eqn. A25-A26). Eqn. A13 summates regional internal logistics costs.

Market Module

$$R_{i,g} \leq A_{i,g} \quad \forall i \in \mathrm{I}, g \in G$$

Eqn.A14

$$D_{i,g} \leq DM_{i,g} \quad \forall g \in G, i \in I$$

Eqn.A15

$$D_{i,g} = DM_{i,g} \quad \forall g \in G, i \in I^{D}$$

Eqn.A16

$$CPC = \sum_{i} \sum_{g} R_{i,g} C_{i}$$

Eqn.A17

$$REV = \sum_{i} \sum_{g} D_{i,g} C_{i}$$

Eqn.A18

Eqn. A14 constrains regional commodity purchase to regional availability. Eqn. A15 similarly constrains commodity sale to regional demand. Eqn. A16 represents the demand pull equality constraint, a key system driver in the absence of profitable operation. Eqn. A17-A18 summate regional commodity purchase costs and sales revenue repectively.

Capacity Module

$$\sum_j \delta_{j,p,g} \leq 1 \quad \forall p \in \mathrm{P}, g \in G$$

Eqn.A19

$$\delta_{j,p,g}.VB_{p,j-1} \leq VI_{j,p,g} \leq \delta_{j,p,g}.VB_{p,j} \quad \forall j \in J, p \in \mathrm{P}, g \in G$$

Eqn.A20

$$V_{p,g} = \sum_j VI_{j,p,g} \quad \forall p \in \mathrm{P}, g \in G$$

Eqn.A21

$$CV_{p,g} = \sum_j \left[\delta_{j,p,g}.CB_{p,j-1} + \left(VI_{j,p,g} - \delta_{j,p,g}.VB_{p,j-1}\right).\left(\frac{VB_{p,j} - VB_{p,j-1}}{CB_{p,j} - CB_{p,j-1}}\right)\right] \quad \forall p \in \mathrm{P}, g \in G$$

Eqn.A22

$$PCC = \sum_p \sum_g CV_{p,g}$$

Eqn.A23

The capacity of each technology within each region is sized and costed with economies of scale through interpolation of a piecewise linear capital cost curve. Eqn. A19 constrains capacity selection to a single scale (i.e. piecewise linear region). Eqn. A20 constrains the capacity relative to the upper and lower bounds for the selected scale. Eqn. A21 summates the selected scale capacity to represent the total regional capacity. Eqn. A22 interpolates the linearised cost-curve for each scale in order to determine the total capital cost for the technology in each region. Eqn. A23 summates the regional capital costs.

Mass Balance Module

$$\sum_{k}\sum_{m}\left(Q_{i,m,k,g}-Q_{i,m,g,k}\right)+\sum_{p}V_{p,g}.\left(\overline{\rho}_{i,p}-\rho_{i,p}\right)+R_{i,g}-D_{i,g}=0 \qquad \forall i\in I, g\in G$$

Eqn.A24

Eqn. A24 represents a mass balance for each commodity within each region. This balance links logistics, production, consumption, purchase and sale.

Objective Function

$$Minimise\left[TOC+TCC+PCC+CPC+TCIT\right]$$

Eqn.A25

$$Maximise\left[REV-\left(TOC+TCC+PCC+CPC+TCIT\right)\right]$$

Eqn.A26

Eqn. A25 represents a standard cost minimisation objective. This can be substituted for Eqn. A26, representing a profit maximisation objective.
